# Role of endometrial microRNAs in repeated implantation failure (mini-review)

**DOI:** 10.3389/fcell.2022.936173

**Published:** 2022-08-19

**Authors:** Sepide Goharitaban, Ali Abedelahi, Kobra Hamdi, Mozafar Khazaei, Masoumeh Esmaeilivand, Behrooz Niknafs

**Affiliations:** ^1^ Immunology Research Center, Tabriz University of Medical Sciences, Tabriz, Iran; ^2^ Department of Anatomical Sciences, Faculty of Medicine, Tabriz University of Medical Science, Tabriz, Iran; ^3^ Womens Reproductive Health Research Center, Tabriz University of Medical Sciences, Tabriz, Iran; ^4^ Fertility and Infertility Research Center, Kermanshah University of Medical Sciences, Kermanshah, Iran; ^5^ Department of Reproductive Biology, Faculty of Advanced Medical Sciences, Tabriz University of Medical Sciences, Tabriz, Iran

**Keywords:** miRNAs, art, transcriptome, pregnancy, endometrial receptivity

## Abstract

MicroRNAs (miRNAs) play various roles in the implantation and pregnancy process. Abnormal regulation of miRNAs leads to reproductive disorders such as repeated implantation failure (RIF). During the window of implantation, different miRNAs are released from the endometrium, which can potentially reflect the status of the endometrium for *in vitro* fertilization (IVF). The focus of this review is to determine whether endometrial miRNAs may be utilized as noninvasive biomarkers to predict the ability of endometrium to implant and provide live birth during IVF cycles. The levels of certain miRNAs in the endometrium have been linked to implantation potential and pregnancy outcomes in previous studies. Endometrial miRNAs could be employed as non-invasive biomarkers in the assisted reproductive technology (ART) cycle to determine the optimal time for implantation. Few human studies have evaluated the association between ART outcomes and endometrial miRNAs in RIF patients. This review may pave the way for more miRNA transcriptomic studies on human endometrium and introduce a specific miRNA profile as a multivariable prediction model for choosing the optimal time in the IVF cycle.

## 1 Introduction

Implantation is a process in which the blastocyst attaches to and attacks the mother’s endometrium within the time frame of the implantation window (Achache and Revel, 2006). The receptive endometrium plays an active role in the implantation process (Kliman and Frankfurter, 2019). In response to steroid hormones, the endometrial tissue undergoes morphological, cellular, and molecular changes in cycles (Davidson and Coward, 2016; Kliman and Frankfurter, 2019). Reproductive specialists currently use methods such as transvaginal ultrasound and hormonal analysis of serum to predict endometrial receptivity for embryo transfer (ET). However, these methods do not provide beneficial predictions for the outcome of *in vitro* fertilization (IVF) (Craciunas et al., 2019; Horcajadas et al., 2008; Quinn and Casper, 2009). Taking into account the molecular changes of the endometrium during the implantation window provides us with crucial information regarding endometrial receptivity, which is of great importance (Craciunas et al., 2019).

Thus far, a range of single molecules, including miRNAs, have been examined as biomarkers of uterine receptivity (Edgell et al., 2013). Reproductive disorders such as polycystic ovary syndrome (PCOS), repeated implantation failure (RIF), and endometriosis are linked to abnormal miRNA regulation (Liang et al., 2017; Shokrzadeh et al., 2018). About 15%–20% of infertile couples who undergo IVF-ET suffer from Repeated implantation failure (RIF) (Simon and Laufer, 2012). About 15%–20% of infertile couples who undergo IVF-ET suffer from RIF. MiRNAs might have the capacity to predict RIF. The present review may help to identify various biomarkers through miRNA detection.

## 2 Repeated implantation failure

**FIGURE 1:**
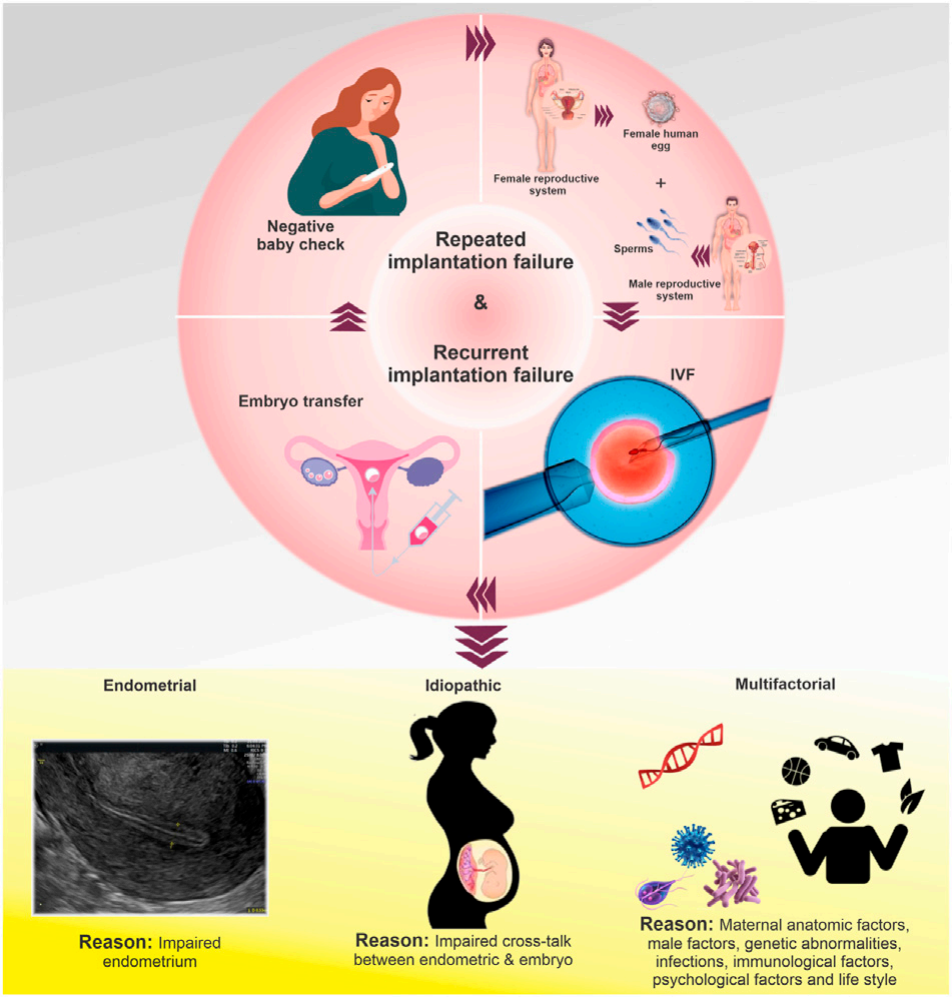
Types of RIF and their causes. The failure of an embryo to implant into the uterine wall after multiple transfers during IVF treatment is referred to as RIF or recurrent implantation failure. RIF types are divided into three categories: Endometrial RIF, which occurs due to the low thickness (≤6 mm) of the endometrium; idiopathic RIF, which is unexplained failure to achieve pregnancy after the transfer of good quality embryos; multifactorial RIF, which is caused by a wide variety of reasons (male-related factors, genetic abnormalities, infections, immunological factors, psychological factors, lifestyle, and other similar variables).

The inability of an embryo to implant into the uterine wall after multiple transfers during IVF treatment is referred to as RIF. However, due to the lack of a unified definition, various definitions of RIF are proposed in IVF centers. RIF is defined by some sources as the non-implantation of embryos in three consecutive cycles with the transfer of up to three high-quality embryos in each cycle, taking into account the number of embryos transferred in each cycle and the IVF success rate (Shufaro and Schenker, 2011). In some centers, the absence of a sac approximately 45 days (week 5 onwards) after the transfer of at least three embryos or the transfer of more than 10 embryos in multiple transfers is considered RIF (Salehpour et al., 2016). The incidence and prevalence of RIF are rarely reported due to the various definitions of this condition (Table 1) (Maesawa et al., 2015). Various types of RIF are classified into three broad categories (Timeva et al., 2014), including endometrial RIF, idiopathic RIF, and multifactorial RIF (Figure 1). The main causes for this complication are fetal defects, decreased uterine receptivity, abnormal anatomy of the uterus, and the medical condition of the mother (Margalioth et al., 2006). Other factors influencing RIF include chromosomal and uterine abnormalities, hormonal and placental disorders, smoking, certain medications, maternal heart and kidney disease, and the quality of the transferred embryo (Coughlan et al., 2014).

**TABLE 1:** Definition of RIF.

References	Definition
Choi et al. (2016)	Failure to conceive in at least three previous IVF cycles with good quality embryos
Revel et al. (2011)	At least four ART cycles with embryo transfer failure
Shi et al. (2017)	Three embryo transfer failures in which at least four morphologically high-grade embryos were transferred
Rekker et al. (2018)	Three unsuccessful IVF cycles with embryo transfer
Xu et al. (2019)	Three transplantation failures with at least four good-quality embryos
Tan et al. (2005)	Non-pregnancy after transfer of more than 10 high-quality embryos, after 2–6 IVF cycles

Thus far, RIF has been managed in a variety of ways, but no consensus has emerged on the most effective method. Some of the RIF-management methods are blastocyst transfer, assisted hatching, co-culture system, sequential transfer, hysteroscopy, endometrial scratching, salpingectomy for tubal disease, extra number embryo transfer, natural cycle, oocyte donation, intra-tubal ET, immune therapy, and endometrial receptivity array (ERA) (Choi et al., 2016; Katzorke et al., 2016).

## 3 Relationship between hormone balance and microRNAs in repeated implantation failure

The human endometrium undergoes cyclical changes due to sex steroid hormones (Klinge, 2012). Previous studies have examined the relationship between sex steroid hormones and miRNAs at different stages of the menstrual cycle. According to these studies, some endometrial miRNAs regulate the expression of estrogen and progesterone, and on the other hand, estrogen and progesterone are involved in regulating the expression of some endometrial miRNAs. The effect of sex steroid hormones on the expression of endometrial miRNAs in animal models such as zebrafish (Cohen et al., 2008) as well as humans (Klinge, 2012) has been investigated. Reed et al. (2018) reported increased expression of miR-181b and let-7e, and decreased expression of mi-R27b in cultured human endometrial stromal cells exposed to estradiol. Similarly, the induction of miR-125b and miR-133a expression has been reported in the cell culture of human endometrial epithelial cells (Chen et al., 2016; Pan et al., 2017). At the time of ovulation, women with high blood progesterone levels under the ovulation stimulation protocol had a higher endometrial expression of miR-30b, miR-125b, miR-424, and miR-451 than women with low blood progesterone levels (Li et al., 2011). Another study compared the expression levels of several miRNAs in the mid-secretory and late proliferative phases of human endometrial epithelial cells. This study indicated that miR-29b, miR-29c, miR-30b, miR-30d, miR-31, miR-193a-3p, miR-200c, miR-203, miR-204, miR-210, miR-345, and miR-582-5p levels were higher in the mid-secretory phase compared to the late proliferative phase. On the contrary, the expression of miR-105, miR-127, miR-134, miR-214, miR-222, miR-369-5p, miR-370, miR-376a, miR-382, miR450, miR-503, and miR-542-3p was lower in the mid-secretory phase compared to the late proliferative phase (Kuokkanen et al., 2010).

The actions of estrogen and progesterone are related to the altered expression of their receptors in the human endometrium. There is not much information about the regulation of estrogen receptors by miRNAs in the human endometrium. However, one study reported that endometrial cancer cells transfected with a miR-107 mimic had lower estrogen receptor expression (Bao W et al., 2019). In addition, miR-22-5p transfection in endometrial stromal cells of female endometriosis in the culture medium altered the estrogen receptor expression (Xiao et al., 2020). Moreover, miR-194-3p transfection in cultured endometrial stromal cells resulted in a significant reduction in progesterone receptor protein levels (PR-A and PR-B) (Pei et al., 2018). Zhou et al. (2016) reported a decrease in protein levels of PR-A and PR-B in endometrial stromal cells transfected with miR-196a (Zhou et al., 2016). They also identified PRs as targets for miR-196a, miR-297, miR-575, miR-628-3p, miR-635, miR-921, miR-938, and miR-1184. It should be noted that miR-92a transfection in the endometrial stromal cell line resulted in progesterone resistance and increased cell proliferation (Zhou et al., 2016).

## 4 The role of endometrial microRNAs in repeated implantation failure

**FIGURE 2:**
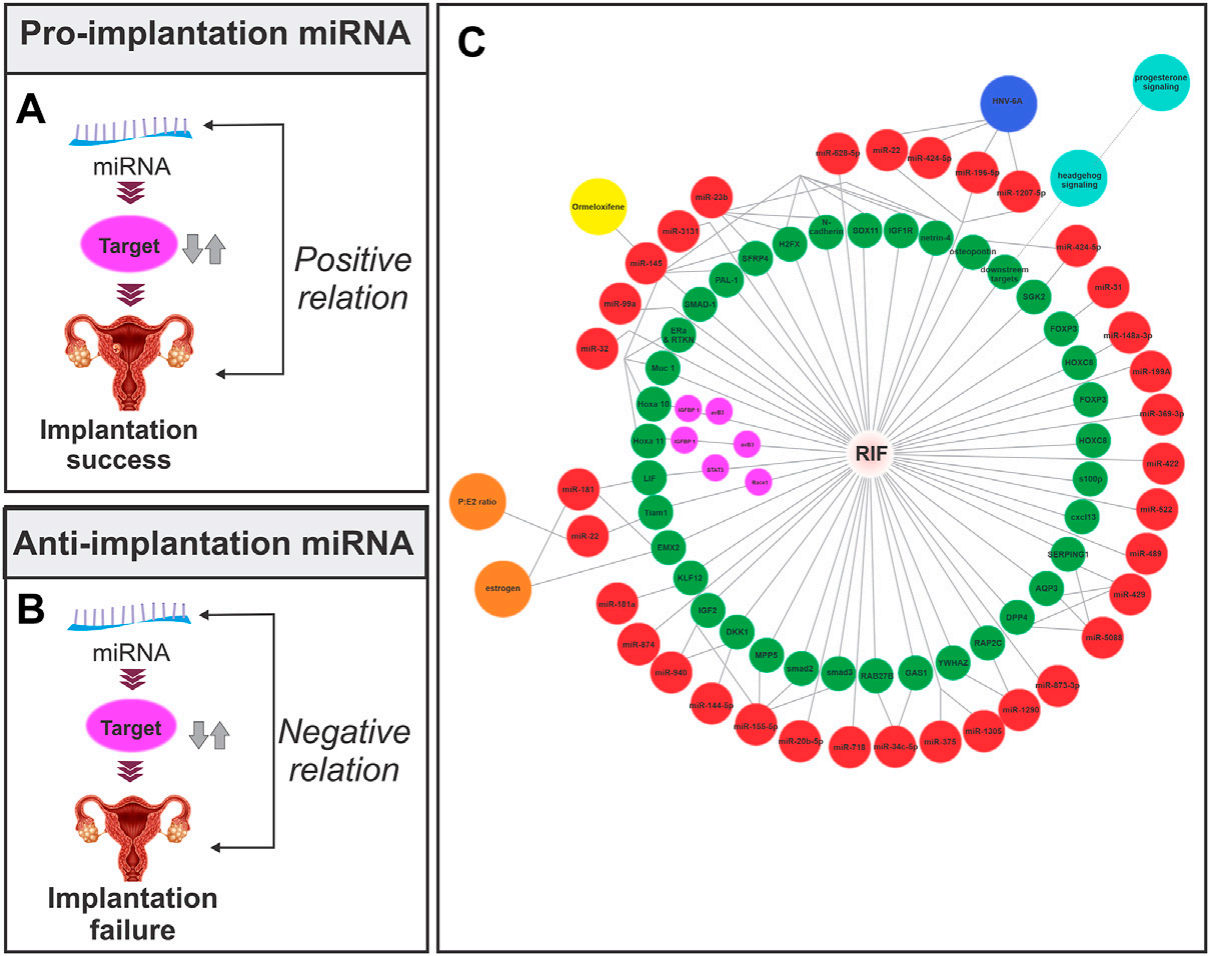
Relationship between the endometrial miRNA expression and implantation. **(A)** Pro-implantation miRNA. The expression of the miRNA has a positive association with the implantation outcome. **(B)** Anti-implantation miRNA. The expression of the miRNA has a negative association with the implantation outcome. **(C)** Endometrial miRNAs lead to implantation failure through their effect on target mRNA in the endometrial tissue of RIF patients. The red circle represents miRNA, the green and purple circles represent mRNA, the orange circle represents hormones, the yellow circle represents the drug, the blue circle represents the signaling pathway, and the navy blue circle represents infection. The miRNA-mRNA network is based on Section 4in this article.

According to the literature, the expression of miRNAs varies in different phases and pathological conditions of the endometrium (Shariati et al., 2019; Shokrzadeh et al., 2019) It has been established that the upregulation of certain miRNAs promotes implantation (pro-implantation miRNAs) while the upregulation of others causes implantation failure (anti-implantation miRNAs) (Reza et al., 2019). MiRNAs that are involved in implantation can also be classified based on their roles into categories such as proliferation, decidualization, angiogenesis, and apoptosis, among others (Table 2) (Figures 2A,B).

**TABLE 2:** Endometrial miRNAs and implantation.

miRNA	Anti/pro implantation	Target gene	Role	Species	References
miR-17–92	Pro-implantation	E2Fs, TGFβ	Decidualization	-	Mogilyansky and Rigoutsos (2013)
miR-21	Pro-implantation	RECK, MMP9, PTEN	Proliferation	Mice	Hu et al. (2008)
miR-21	Pro-implantation	KLF12	Decidualization	Human	Jiang et al. (2013)
miR-29a	Pro-implantation	Bak1, Bmf, Bcl-w	Apoptosis	Rat	Xia et al. (2014a)
miR-101a	Pro-implantation	Cox-2	Decidualization	Mice	Chakrabarty et al. (2007)
miR-199a	Pro-implantation	Cox-2	Decidualization	Mice	Chakrabarty et al. (2007)
miR-199a	Pro-implantation	Muc1	Decidualization	Mice	Inyawilert et al. (2014)
miR-199a	Pro-implantation	Grb10	Proliferation and apoptosis	Rat	Xia et al. (2014b)
miR-22	Anti-implantation	Tiam1, Rac1	Cell migration-motility	Mice	Ma et al. (2015)
Let-7	Pro-implantation	Muc-1	Inhibits proliferation-promotes differentiation	Mice	Inyawilert et al. (2015)
miR-200	Anti-implantation	Zeb1, Zeb2, PTEN	Proliferation and apoptosis	Mice	Jimenez et al. (2016)
miR-30d	Pro-implantation	H19, NNMT	Proliferation, hormonal responses, methylation status	Human	Moreno-Moya et al. (2014)
miR-98	Anti-implantation	Bcl-xL	Promotes proliferation and inhibits apoptosis	Rat	Xia et al. (2014c)
miR-141	Anti-implantation	PTEN	Proliferation, apoptosis	Mice	Liu et al. (2013)
miR-143	Pro-implantation	LIFR	Proliferation, invasion, decidualization	Rat	Tian et al. (2015)
miR-181a	Pro-implantation	KLF19	Decidualization, differentiation	Human	Zhang et al. (2015)
miR-193	Pro-implantation	GRB7	Migration	Mice	Li et al. (2014)
miR-429	Anti-implantation	Pcdh8	Invasion	Mice	Li et al. (2015a)
miR-451	Pro-implantation	Ankrd46	Angiogenesis, invasion, and proliferation	Mice	Li et al. (2015b)
miR-222	Anti-implantation	CDKN1C, p57kip2	Differentiation cell cycle, decidualization	Human	Qian et al. (2009)
miR-424	Anti-implantation	-	-	Human	Li et al. (2011)
miR-30b	Pro implantation	P4HA2	-	Human	Li et al. (2011)
miR-125b	Anti-implantation	MMP26	Migration and invasion	Mice	Li et al. (2011)

The first study to investigate the different expressions of miRNAs in the secretory phase endometrium of RIF patients was published in 2011, and it found 13 miRNAs that could be used to diagnose and treat RIF (Revel et al., 2011). Since then, many human studies have found different expressions of several miRNAs in the endometrial tissue or peripheral blood of women with RIF (Figure 2C). These studies have shown that the profile of miRNAs in the pre-receptive and receptive endometrium of RIF patients is different from that of normal individuals, indicating the role of miRNAs in the implantation failure in these patients. The following is an overview of some RIF-related miRNAs (Table 3).

**TABLE 3:** Endometrial miRNAs and repeated implantation failure.

Year	Sample	Group compare	miRNA measurement	miR	Expression pattern	Targets/regulators	References
2011	EB	Receptive endometrium in RIF patients vs. fertile patients	TaqMan	miR-23b, miR-145, miR-99a, miR-27b, miR 652, miR-139-5p, miR-195, miR-342-3p, miR-150, miR-374b and miR-32, miR-628-5p, miR-874	Upregulated in RIF, and Downregulated in RIF	N-cadherin, H2AFX, Netrin-4, SFRP4	Revel et al. (2011)
2015	EB	Infertile patients with RIF, vs. control group	Real-Time PCR	miR-22	Upregulated in RIF	Tiam1/Rac1	Ma et al. (2015)
2015	EB	Women with a normal menstrual cycle	-	miR-181a	-	KLF12	Zhang et al. (2015)
2016	EB	Healthy volunteers and RIF	miRNA microarrays	miR-138-1-3p, miR-29b-1-5p, miR-363-3p, miR-34b-3p, miR-146a-5p, miR-363-3p	Upregulated in RIF	S100P, CXCL13, SIX1	Choi et al. (2016)
2017	EB	Receptive endometrium in RIF patients vs. infertile patients	miRNA Complete labeling	miR-1207-5p, miR-4306, miR-572, miR-5739, miR-30b, miR-6088 and miR-374a-5p, miR-145-5p, miR-196b-5p, miR-199a-5p, miR-449a, miR-424-5p, miR-125b-5p,miR-21-5p	Downregulated in RIF and Upregulated in RIF	ERα, RTKN	Shi et al. (2017)
2018	Blood, EB	Fertile and RIF patients	Small RNA sequencing	miR-30b-5p, miR-30d-3p, miR-30d-5p, miR-30a-5p	Dysregulation	CDK5, STAT3	Rekker et al. (2018)
2019	Blood	RIF in women with or without metabolic syndrome	Real‐Time PCR	miR‐21, miR‐223, miR-146a	Increased in RIF‐MS patients, declined in RIF‐MS patients	-	Sheikhansari et al. (2019)
2019	EB	RIF and healthy female controls	-	miR-489, miR-199A, miR-522, miR-369-3p, miR-422	Considered as the key regulatory factors during RIF	UBE2I, PLK4, XPO1, AURKB, NUP107, E2F4, SIN3A	Wang and Liu (2020)
2020	EB	Normal fertile women and RIF	Real‐Time PCR	miR-31	Increase in RIF	FOXP3	Dehghan and Salehi (2020)
2020	EB	RIF	RT-qPCR	miR-152-3p, miR-155-5p, miR-455-3p, miR-4423-3p	Overexpression	-	Drissennek et al. (2020)
2020	EB	Women with and without RIF	Microarray, RT-qPCR	miR-148a-3p	Upregulated in RIF	HOXA8	Zhang et al. (2020)
2020	Human endometrial HEC-1A cell	-	Microarray	miR-15, miR-22, miR-196-5p and miR-1207-5p	Upregulated in RIF, and Downregulated in RIF	-	Bortolotti et al. (2020)
2020	EB	RIF, control group	RT-qPCR	miR-145	Upregulated in RIF	PAI-1	Liu et al. (2020b)

### 4.1 MiR-145

MiR-145 was first discovered in 2001 on chromosome 18 in mice, and then in 2003, on chromosome 5 in humans (Yuan et al., 2019). It is abundant in mesoderm-derived tissues, such as the uterus, ovaries, testes, prostate, and heart, and plays a key role in endometrial differentiation. (Chang et al., 2017). This miRNA inhibits the SMAD-1 pathway, angiogenesis, and stromal cell differentiation while also regulating decidua cell proliferation (Sirohi et al., 2019). MiR-145 shows a threefold increase in RIF patients compared to fertile individuals (Liu et al., 2020a). Targets of this miRNA include insulin-like growth factor 1 receptor (IGF1R), rhotekin (RTKN), estrogen receptor alpha (Era), octamer-binding transcription factor 4 (OCT4), SRY-related HMG-box 11 (SOX11), mucin 1 (Muc1), PAL-1, homeobox A10 (HOXA10), and homeobox A11 (HOXA11). The attachment of the mouse embryo to the endometrium is inhibited during an increase in miR-145 levels or a decrease in IGF1R levels in endometrial epithelial cells (Kang et al., 2015). The expression of miR-145 in the endometrium of mice treated with ormeloxifene, which is a non-steroidal oral medication used to prevent endometrial receptivity on the first day of pregnancy, is increased (Sirohi et al., 2018). Moreover, miR-145 affects receptivity and implantation by targeting PAL-1 and reducing its expression in the endometrium of RIF patients (Liu et al., 2020a). This miRNA inhibits SOX11 in endometrial cancer and suppresses the proliferation, migration, and invasion of HCC-1 cell lines while increasing the induction of apoptosis. In addition, by inhibiting OCT4, it also prevents the growth of endometrial cancer cells (Chang et al., 2017). Elevated miR-145 expression inhibits HOXA10 and HOXA11. Inhibition of these genes (*HOXA10* and *HOXA11*) by acting on IGFBP-1 and avB3 leads to infertility (Nazarian et al., 2022).

### 4.2 MiR-22

MiR-22 is an anti-implantation miRNA whose expression is increased during the normal cycle window of implantation in RIF patients. MiR-22 leads to the dysregulation of decidualization in endometrial stromal cells by targeting Tiam1. Tiam1 with the help of Race1 is involved in stromal cell decidualization, uterine receptivity regulation, migration, and implantation (Grewal et al., 2008). An increased expression of miR-22 leads to a decreased expression of Tiam1. As a result, the reduction of Tiam1/Race1 signaling will lead to implantation failure in RIF patients (Ma et al., 2015). The abnormal expression of miR-22 and Tiam1/Race1 has been linked to a decrease in the progesterone/estradiol (P/E2) ratio in RIF patients. Transfection of miR-22 in cultured stromal cells isolated from the endometrium of female endometriosis leads to changes in estrogen receptor (ER) expression. MiR-22 suppresses the estrogen signaling pathway by targeting estrogen receptor 1 (ESR1), which is essential for the formation of the male glands (Xiao et al., 2020; Li et al., 2021; Shekibi et al., 2022).

### 4.3 MiR-181

The expression of miR-181 is reduced in RIF patients (Chu et al., 2015). Leukemia inhibitory factor (LIF) and Kruppel-like factor 12 (KLF12) are the targets of miR-181. Estrogen has been shown to regulate empty spiracles homeobox 2 (EMX2) levels, which in turn control miR-181 expression. In fact, estrogen reduces the expression of EMX2 and EMX2 suppresses the expression of miR-181 (Troy et al., 2003). Subsequently, the reduction of miR-181 expression leads to a rise in LIF levels, resulting in implantation success. LIF is a proinflammatory cytokine from the interleukin 6 (IL-6) family that plays an important role in preparing the uterus for embryo implantation (Aghajanova et al., 2009). Mariee et al. (2012) reported decreased LIF expression in RIF patients. MiR-181a is a member of the miR-181 family whose expression is suppressed by estrogen. MiR-181a also inhibits the expression of KLF12, which is required for endometrial receptivity. It has been reported that KLF12 expression is increased in the endometrium of RIF and endometriosis patients (Maillot et al., 2009; Zhang et al., 2015).

### 4.4 MiR-424-5p

MiR-424-5p can be a useful marker in assessing endometrial receptivity. The expression of this miRNA increases in the endometrium of RIF patients (Rekker et al., 2018). Moreover, decreased miR-424-5p expression has been reported in the mid-secretory endometrium of fertile women (Rekker et al., 2018). The targets of miR-424-5p include the secreted phosphoprotein 1 (*SPP1*), serum/glucocorticoid regulated kinase 2 (*SGK2*), and angiogenin (*Ang*) genes. Osteopontin is a glycoprotein encoded by *SPP1* and its expression in the endometrium is associated with infertility. MiR-424-5p targets osteopontin and thus regulates adhesion and cell migration during implantation (Johnson et al., 2014; Kang et al., 2014). Progesterone also regulates osteopontin expression during the endometrial menstrual cycle (Casals et al., 2010). SGK2 is a protein kinase, that is, involved in cell proliferation as well as the regulation of endometrial receptivity by acting on ion channels (Gamper et al., 2002). In addition, *Ang* is a gene, that is, regulated by miR-424-5p and encodes the vascular endothelial growth factor (VEGF) protein. MiR-424-5p expression is reduced in tissues with high progesterone levels compared to those with normal progesterone levels. The role of miR-424-5p in cancer has also been reported, which, given the miR-424-5p targets listed above, could introduce a common molecular pathway between implantation and cancer (Kolanska et al., 2021).

### 4.5 MiR-155-5p

MiR-155-5p expression is increased in RIF patients (Chen P et al., 2021; Drissennek et al., 2022). MiR-155-5p is involved in implantation by targeting genes such as membrane palmitoylated protein 5 (*MPP5*), insulin-like growth factor 2 (*IGF2*), and transforming growth factor beta (*TGFβ*). TGFβ is involved in leukocyte extravasation signaling, which has been reported to play a role in implantation. Alteration in miR-155-5p expression contributes to implantation failure because it leads to the inhibition of smad2/3 as well as the suppression of essential processes in implantation (cell proliferation, migration, apoptosis, and invasion) (Lin et al., 2018; Chen et al., 2019; Luo et al., 2020). Because smad2/3 is one of the most important genes in TGFβ signaling, miR-155-5p may be involved in implantation failure by altering this pathway (Drissennek et al., 2022). MiR-155-5p affects the function of the MPP5 protein in the menstrual cycle of RIF patients. MPP5 expression in the normal menstrual cycle gradually decreases from the beginning of the proliferation stage to the end of the secretory stage (Li et al., 2017). IGF2 is another protein whose expression is greatly increased during the implantation window and miR-155-5p suppresses the expression of this protein (Whitby et al., 2018).

### 4.6 MiR-31

MiR-31 is a candidate for endometrial receptivity whose expression is increased in RIF patients compared to healthy women (Shi et al., 2017). MiR-31 expression is increased in the secretory phase of fertile women compared to the proliferative phase (Azhari et al., 2022). Moreover, miR-31 expression is increased in the serum of fertile patients in the secretory phase. MiR-31 expression is also reduced in the endometrial secretory phase of RIF patients compared to the proliferative phase. In addition, miR-31 expression is increased in the early secretory stage compared to the midluteal stage in the endometrium of women with regular menstrual cycles. Considering the above, miR-31 plays an important role in implantation (Ghafouri-Fard et al., 2021; Kresowik et al., 2014). MiR-31 target genes include forkhead box P3 (*FOXP3*) and C-X-C motif chemokine 12 (*CXCL12*), which are suppressed by miR-31 in the secretory phase of fertile women (Azhari et al., 2022). Increased expression of miR-31 leads to decreased expression of FOXP3 (immune suppressor), which can be considered the reason for implantation failure and recurrent miscarriage in RIF patients (Azarpoor et al., 2020). Another target of this miRNA is MMPs, which are involved in decidualization during implantation, defense mechanisms, and immune responses (Azhari et al., 2022).

### 4.7 MiR-34c-5p

Another miRNA of interest in RIF is miR-34c-5p (Tan et al., 2020). This miRNA is involved in endometrial receptivity and inflammation (Cai et al., 2018; Gao et al., 2019). An increase in the levels of this miRNA leads to a decrease in *GAS1* during implantation, which results in implantation failure. Thus, miR-34c-5p is negatively associated with implantation. Tan et al. found an increase in miR-34c-5p in exosomes, indicating that endometrial receptivity-associated miRNAs are present in the small extracellular vesicles (sEVs) of uterine fluid. During endometrial implantation, miR-34c-5p is increased in these vesicles to suppress *RAB27B* and it is simultaneously decreased in the endometrium; therefore, miR-34c-5p levels in sEVs can be used as a marker to assess the physiological condition of the uterus and confirm the most appropriate time for implantation (Tan et al., 2020).

### 4.8 MiR-1290

MiR-1290 expression is increased in the endometrium of RIF patients (Liu et al., 2021). An elevated expression of this miRNA has also been observed in the endometrial extracellular vesicles of RIF patients (Ponsuksili et al., 2014). MiR-1290 has an inhibitory role in endometrial cell proliferation, and *YWHAZ* and *RAP2C* are the targets of this miRNA. MiR-1290 reduces *YWHAZ* in RIF patients. An increase in miR-375 and miR-1305 has also been reported in RIF (Luo et al., 2021).

### 4.9 MiR-148a-3p

The *HOX* genes, specifically the *HOXA10* and *HOXA11* genes, have an important role in implantation (Cakmak and Taylor, 2011). In addition, *HOXC8* is involved in cell proliferation, differentiation, migration, adhesion, and tumorigenesis (Liu et al., 2018). *HOXC8* is introduced as a target for miR-148a-3p (Zhang et al., 2020). With the increase of miR-148a-3p (miR-148/152 family) in RIF patients, *HOXC8* is suppressed, which leads to implantation failure due to reduced decidualization. According to Chen et al. (2013), miR-148a-3p also suppresses tumors and is involved in various processes such as differentiation and development (Choi et al., 2016; Zhang et al., 2020).

### 4.10 MiR-21 and MiR‐146a

Given the increase in inflammatory miRNAs and cytokines in RIF, Sheikhansari et al. (2019) reported that the expression of miR-21 was increased in the RIF-metabolic syndrome (MS) group and the expression of miR-223 and miR-146a was reduced in this group. The reduction of miR‐146a leads to an increase in inflammatory factors and the inhibition of the IRAK1‐TRAF6‐NF‐κB pathway (Ghaebi et al., 2019). The function of miR‐21 can lead to inflammatory responses, suppression of the immune system, or stimulation of inflammation by inhibiting the TGFβ signaling pathway (Sheedy, 2015).

### 4.11 MiR-135b-5p

MiR-135b-5p is increased in the endometrium of RIF patients compared to healthy women (Shang et al., 2022). The targets of miR-135b-5p are podoplanin (*PDPN*) and angiotensinogen (*AGT*) (Shang et al., 2022). The reduction of miR-135b-5p expression plays a role in increasing the decidualization of endometrial stromal cells (Wang et al., 2021). Moreover, this miRNA is introduced as a biomarker in breast cancer due to its role in proliferation and migration (Bao C et al., 2019).

### 4.12 Other microRNAs

In addition to the miRNAs mentioned above, a number of miRNAs have been introduced in the literature, the expression of which is different in RIF patients at different stages or when compared to the control group. However, further validation and determination of specificity, sensitivity, and accuracy are required before these miRNAs can be studied in RIF patients. For this purpose, we merely covered basic information on these miRNAs in this section.

Shi et al. (2017) found a decrease in miR-4668-5p expression and an increase in miR-429, miR-5088, and miR-374 expressions in the RIF group (Shi et al., 2017). MiR-374 is involved in implantation by activating Wnt/β-catenin signaling. RIF patients also have lower levels of targets associated with miR-429 and miR-5088, which include dipeptidyl peptidase-4 (*DPP4*), *SERPING1*, and aquaporin 3 (*AQP3*). An increase in miR-30b expression is not associated with RIF since this miRNA is also overexpressed in the normal endometrium (Shi et al., 2017). MiR-152-3p is another RIF-related miRNA, which suppresses cell proliferation, migration, and angiogenesis (Haouzi et al., 2009; Drissennek et al., 2020).

Other miRNAs associated with RIF include miR-489, miR-199A, miR-369-3p, miR-422, and miR-522. The role of miR-489 has been established in many cancers, and the genes associated with this miRNA include *RBBP6*, *NHS*, *ATRX*, and *XPO1*. Genes associated with miR-199A, which is reduced in endometriosis, include *CTDSPL2*, *HOXA9*, *LUC7L3*, *EML4*, *HYOU1*, and *PDS5B*. Moreover, *UBE2I*, *PLK4*, *XPO1*, *AURKB*, and *NUP107* are other genes involved in cell division and endometrial stromal cell differentiation, which have an elevated or reduced expression in RIF patients. Other associations that affect the function of miRNAs include miRNA-transcription factor (TF) interactions. According to the findings of Wang and Liu (2020), E2F4 and SIN3A are among the TFs effective in RIF, which are linked to genes related to several miRNAs involved in this condition (Wang and Liu, 2020). Furthermore, the *HTR1A*, *NR3C1*, and *GABRA3* genes are essential in determining the medication treatment of RIF. Due to its identified features in RIF, *NR3C1* has attracted the most attention (Tuckerman et al., 2010).

MiR-23a and miR-23b, in conjunction with the long non-coding RNA (lncRNA) PART1, decrease DUSP5 and ultimately inhibit the mitogen-activated protein kinase (MAPK) pathway in RIF patients. In addition, the association of miR-96-5p with the phosphatidylinositol 3-kinase (PI3K)-protein kinase B (Akt) pathway and the *PTEN* gene leads to endometrial abnormalities and affects endometrial receptivity (Chen C. H et al., 2021).

The expressions of miR-138-1-3p, miR-29b-1-5p, miR-363-3p, miR-34b-3p, miR-146a-5p, and miR-363-3p in RIF patients were different (Choi et al., 2016). Finally, it is worth noting that the study of polymorphisms in some miRNAs in RIF patients has gained much attention in recent years (Lee et al., 2020).

## 5 Are microRNAs potential biomarkers of repeated implantation failure detection in assisted reproductive technology?

Finding suitable strategies with acceptable specificity and sensitivity and minimal invasiveness is essential for determining the window of implantation.

The role of miRNAs in regulating biological cascades makes them potent diagnostic biomarkers. MiRNAs obtained from plasma, plasma exosomes, follicular fluid, uterine fluid, and endometrial tissue are presented as diagnostic biomarkers to estimate the window of implantation. Chen P et al. (2021) showed that endometrial-specific miRNAs can be considered diagnostic biomarkers for RIF prediction. They compared endometrial tissue biopsies from RIF and non-RIF patients using ERA technology and miRNA expedition. They identified three miRNAs, including miR-20b-5p, miR-155-5p, and miR-718, which can serve as biomarkers in RIF with 90% accuracy (specificity: 100.0%; sensitivity: 80.0%; positive prediction value: 100.0%; negative prediction value: 85.7%) (Chen P et al., 2021). In another study on miRNAs extracted from plasma and plasma exosomes, miR-150-5p, miR-150-3p, miR-149-5p, and miR-146b-3p were introduced as candidates for non-invasive biomarkers of RIF (Zeng et al., 2021). Moreover, a study on cumulus cells and follicular fluid of the RIF patients’ oocytes identified the overexpression of miR-34-5p and miR-26-5p as an indicator of a successful pregnancy (Habibi et al., 2022). In addition, von Grothusen et al. (2022) reported the decline of the expression of miR-486-5p and miR-92b-3p in the uterine fluid as a non-invasive biomarker of RIF conditions.

**FIGURE 3:**
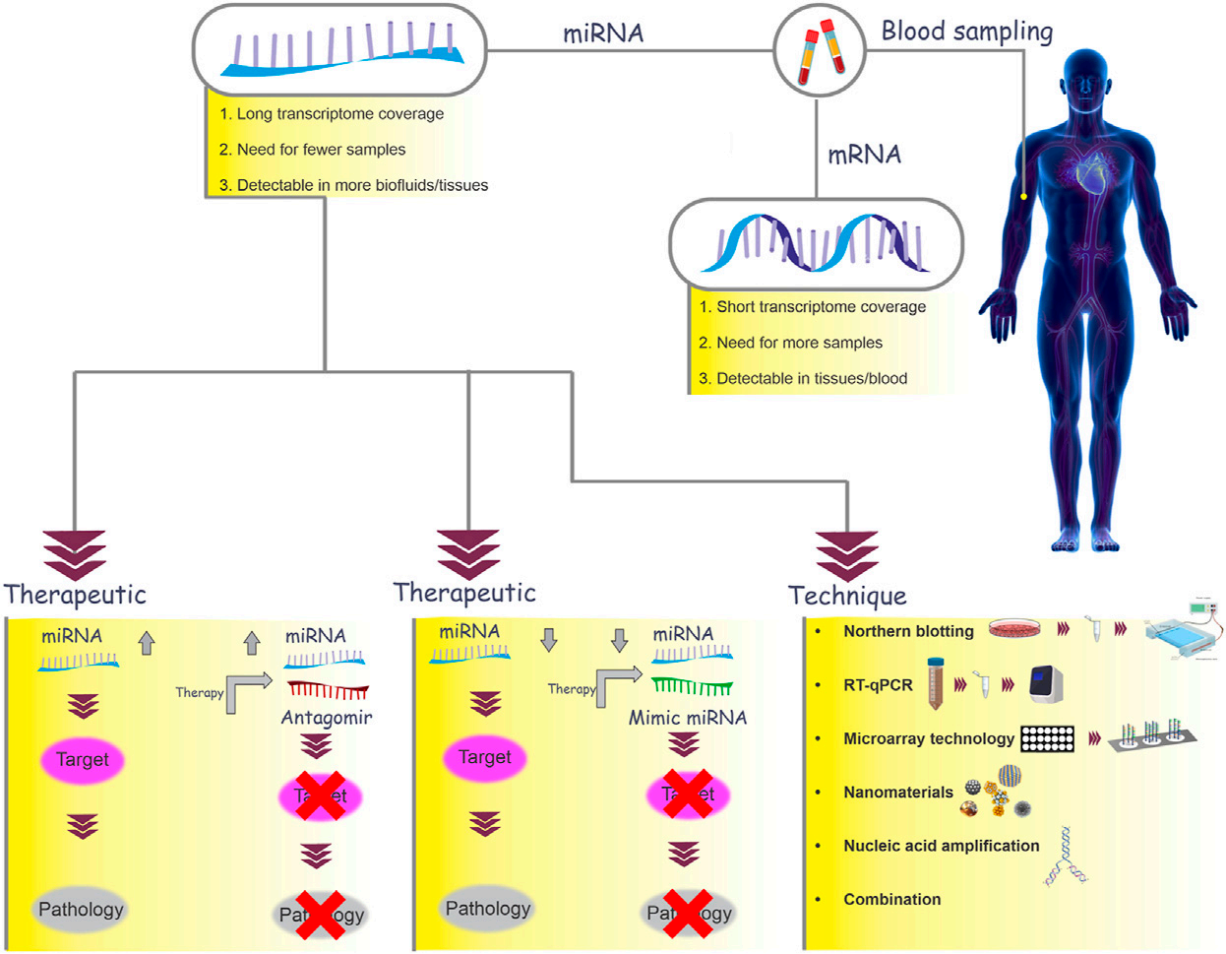
MiRNAs as biomarkers. In this graph, mRNAs and miRNAs are compared as two biomarker candidates. MiRNAs can be used as therapeutic targets in a number of ways. Depending on whether the miRNA is upregulated or downregulated in the disease, there are generally two approaches: miRNA inhibition and miRNA replacement. If an increase in the miRNA expression leads to pathology (e.g., RIF), the use of miRNA antagomirs in therapeutic methods will prevent the binding of miRNAs to the target mRNA and will reduce the symptoms of the disease. On the other hand, if a reduction in miRNA expression leads to pathology, a miRNA delivery system (miRNA mimic) can be used. There are several techniques for detecting miRNAs (northern blotting, reverse transcription quantitative real-time PCR (RT-qPCR), microarray technology, nanomaterial-based methods, and nucleic acid amplification).

In this regard, targeting these miRNAs to either down or upregulate them may be an effective treatment for RIF. Therefore, manipulating miRNA-related signaling in favor of implantation by targeting these miRNAs can be considered a potential therapeutic approach (Figure 3) (Krützfeldt, 2016). Further studies are necessary to introduce miRNAs that can be used to determine the window of implantation time.

## 6 Conclusion and prospects

RIF is a growing problem in the field of reproductive medicine. Repetition of failed cycles in RIF patients is very costly for the patients and affects the physical and psychological health of those under treatment. Determining the expression of miRNAs and key genes in the endometrium can be beneficial in predicting the success rate of implantation in clinics. Since one type of miRNA can affect several target signaling pathways, and subsequently, change the fate of the cell, it is possible to use miRNAs to prevent implant failure or treat RIF patients. Nevertheless, research on the treatment of RIF using miRNA-targeting strategies is still lacking. In this review, we identified three miRNAs that are capable of acting as biomarkers in RIF, namely miR-20b-5p, miR-155-5p, and miR-718. In conclusion, specific endometrial miRNAs are suitable as diagnostic or therapeutic biomarkers in RIF.
