# Supplementary figures and images for: Role of endometrial microRNAs in repeated implantation failure (mini-review)
Source: Front Cell Dev Biol. 2022 Aug 19;10:936173. doi: 10.3389/fcell.2022.936173 (PMC9437697; doi:10.3389/fcell.2022.936173)

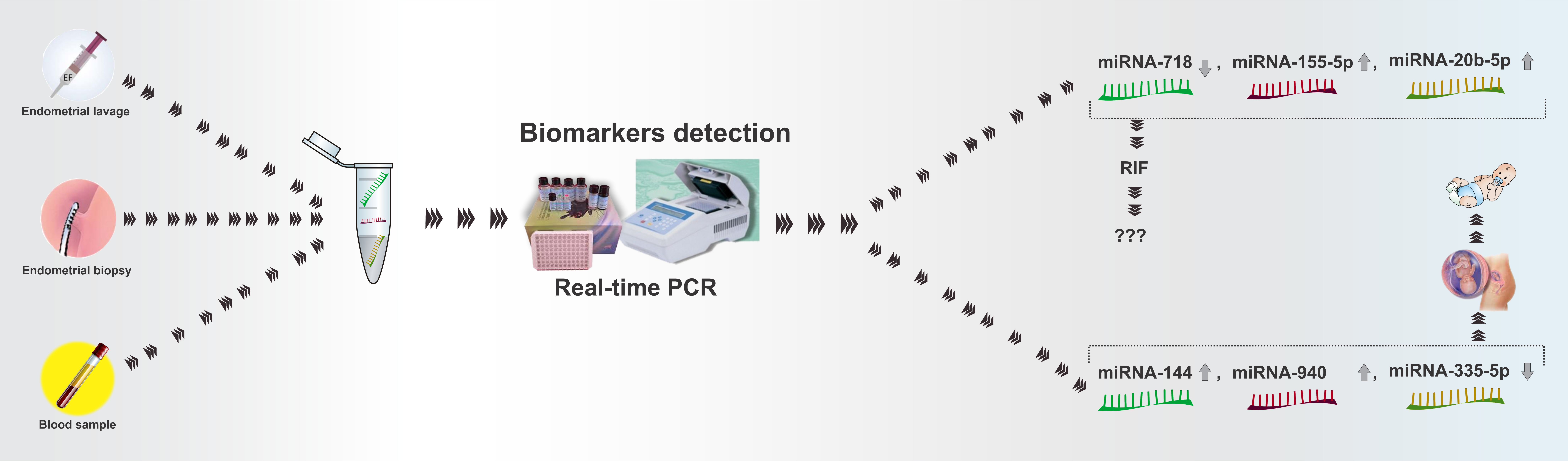

Supplement: Supplementary file 1 [file Image1.JPEG]
